# A Triplex Propidium Monoazide (PMA) qPCR Assay Enables Rapid Discrimination of Live Porcine Reproductive and Respiratory Syndrome Viruses

**DOI:** 10.1155/tbed/7921675

**Published:** 2025-11-07

**Authors:** Xiaoyang Zhu, Wenhao Qi, Hong Lin, Yuan Wang, Yuejia Qiu, Ming Qiu, Meng Cui, Shuai Yang, Yanhan Lin, Yifan Meng, Wanglong Zheng, Jianzhong Zhu, Zeji Lu, Kewei Fan, Nanhua Chen

**Affiliations:** ^1^Preventive Veterinary Medicine, College of Veterinary Medicine, Yangzhou University, Yangzhou 225009, Jiangsu, China; ^2^Kunshan Fourth Animal Epidemic Prevention Station, Kunshan Fourth Animal Health Supervision Branch Institute, Kunshan 215321, Jiangsu, China; ^3^Longyan University and Fujian Provincial Key Laboratory for Prevention and Control of Animal Infectious Diseases and Biotechnology, Longyan 364012, Fujian, China; ^4^Xinjiang TianKang Animal Husbandry Technology Co., Ltd, Changji 831100, Xinjiang, China; ^5^Joint International Research Laboratory of Agriculture and Agri-Product Safety, Yangzhou 225009, Jiangsu, China; ^6^Comparative Medicine Research Institute, Yangzhou University, Yangzhou 225009, Jiangsu, China; ^7^Jiangsu Co-Innovation Center for Prevention and Control of Important Animal Infectious Diseases and Zoonoses, Yangzhou University, Yangzhou 225009, Jiangsu, China

**Keywords:** a triplex PMA-qPCR, discrimination of live viruses, environmental feces, porcine reproductive and respiratory syndrome virus (PRRSV), prevalent PRRSV isolates

## Abstract

The devastating swine disease, porcine reproductive and respiratory syndrome (PRRS), can only be caused by live PRRS virus (PRRSV) infection. However, the most commonly used detection methods cannot discriminate PRRSV infectivity. Here we developed a triplex propidium monoazide (PMA) qPCR assay for differential detection of infectious PRRSV isolates (NADC34-like PRRSV-2, NADC30-like PRRSV-2, and HP-PRRSV-2) prevalent in China. First, the PRRSV inactivation strategy was selected by comparing distinct inactivation methods. Subsequently, we optimized PMA pretreatment parameters and concentrations of primers and probes. The triplex PMA-qPCR assay displayed favorable specificity, sensitivity, and reproducibility. Moreover, 452 clinical samples (environmental feces, lungs, lymph nodes (LNs), and sera) were submitted to differential detection by triplex qPCR and triplex PMA-qPCR assays. A total of 83 PRRSV-positive samples were detected by the triplex qPCR assay, including 25 NADC34-like, 48 NADC30-like, and 15HP-PRRSV-2-positive samples (two samples were coinfected by NADC34-like and NADC30-like PRRSV-2, while three samples were coinfected by NADC30-like and HP-PRRSV-2). Meanwhile, 65 samples were identified by the PMA-qPCR method, including 21 NADC34-like, 36 NADC30-like, and 9HP-PRRSV-2 positive samples (one sample was coinfected by NADC34-like and NADC30-like PRRSV-2). No PRRSV could be isolated from the 18 qPCR-positive but PMA-qPCR-negative samples. Overall, this study provides the first triplex PMA-qPCR assay for rapid discrimination of live PRRSV isolates in clinical samples, particularly in environmental feces.

## 1. Introduction

Porcine reproductive and respiratory syndrome virus (PRRSV) infection costs an estimated $2.7 billion annually in swine industry worldwide [1], which was first identified in Netherlands and North American in 1990s [2, 3]. PRRSV is a single-stranded RNA virus clustering in the *Arteriviridae* family [4]. Due to high genetic diversity, PRRSV isolates are clustered into two species: PRRSV-1 and PRRSV-2 [5]. In China, PRRSV-2 was first identified in 1995 [6]. Since 2006, highly pathogenic PRRSV-2 (HP-PRRSV-2) caused unparalleled outbreaks [7]. From 2013, NADC30-like PRRSV-2 emerged in Chinese swine herds [8]. Since 2017, NADC34-like PRRSV-2 has also been prevalent in China [9]. Nowadays, NADC34-like, NADC30-like, and HP-PRRSV-2 are predominant isolates threatening China's swine industry [10].

Rapid and differential detection of PRRSV is the essential step to control PRRS. Molecular biological assays such as PCR and quantitative PCR (qPCR) targeting viral nucleic acids are the most commonly used methods [11, 12]. Multiplex qRT-PCR assays can simultaneously differentiate distinct PRRSV isolates [13, 14]. The combination of reverse transcription recombinase polymerase amplification (RT-RPA) with CRISPR-Cas13a or a lateral flow dipstick (LFD) enables rapid visual detection of PRRSV [15, 16]. However, they cannot determine whether the viral RNA is from live or inactivated virus. Serological methods may detect PRRSV specific antibodies [17], but they cannot distinguish the antibodies produced by modified live virus (MLV) vaccination or kill vaccine (KV) immunization. Virus isolation is able to determine PRRSV infectivity but it's too complicated [18]. Hence, new methods are urgently needed for infectivity evaluation of prevalent PRRSV isolates.

Propidium monoazide (PMA) combined with PCR has been utilized to distinguish live and inactivated enveloped viruses [19–23]. Mechanistically, when a live enveloped virus was exposed to PMA and light, the PMA dye could not penetrate viral envelope. Therefore, no viral DNA/RNA modification occurred and it could be amplified by PCR. In contrast, when the virus was inactivated and the viral envelope was ruptured, the PMA would covalently bind to DNA/RNA upon exposure to intense visible light, which could prevent the viral DNA/RNA from being amplified by PCR [22]. However, no assay has been developed yet to discriminate live PRRSV isolates prevalent in China. Therefore, PMA pretreatment was integrated with qPCR (PMA-qPCR) to discriminate viral nucleic acids from infectious and noninfectious PRRSV isolates (NADC34-like, NADC30-like, and HP-PRRSV-2) in this study. The efficacy of this new triplex PMA-qPCR method was evaluated via the detection of 452 clinical samples.

## 2. Materials and Methods

### 2.1. Viruses and Cells Used in This Study

The prevalent PRRSV-2 NADC34-like BJ1805-2 isolate, NADC30-like SD17-38 isolate, and HP-PRRSV-2 XJ17-5 isolate were used as representative strains in this study [24, 25]. In addition, PRRSV-1 SD1291 isolate, classical PRRSV-2 VR-2332-like JSYC20-05-1 isolate, CH-1a-like SD1612-1 isolate, and other common porcine viruses, including CSFV JS1805-2 strain, PEDV XM1-2 strain, PDCoV SDLY2302-1721 strain, ASFV Yangzhou strain, PRV XJ03 strain, PCV2 SD17-36 strain, and PPV7 JSYZ20190725-717 strain, were also utilized for the specificity evaluation in this study [22, 25]. Pulmonary alveolar macrophages (PAMs) were prepared as previously described [26].

### 2.2. Clinical Sample Information

A total of 452 clinical samples, including 113 environmental feces, 278 lungs, 37 lymph nodes (LNs), and 24 serum samples, were submitted by farm owners to the Animal Hospital of Yangzhou University from seven regions of China (Jiangsu, Beijing, Fujian, Shandong, Guangdong, Henan, and Sichuan) during April 26th 2020 and February 21st 2025. We were informed that the feces were collected from pig pens and the tissue samples were collected from dead pigs in corresponding pig farms. The detailed infection statuses of these samples were not provided by farm owners. These clinical samples were detected by both triplex qPCR and triplex PMA-qPCR assays.

### 2.3. Inactivating Strategies

PRRSV inactivation was modified from the previous study [22]. Briefly, the same amount (0.1 MOI) of NADC34-like PRRSV-2 BJ1805-2 isolate was used for the inactivation evaluations in three replicates. The virus were inactivated by ultraviolet (UV) light, heat, and three disinfectants, respectively. First, PRRSV samples were disinfected for 10–30 min with a low-pressure mercury vapor lamp emitting monochromatic (254 nm) UV light (Qingdao Haier Biomedical Co. Ltd.). Second, PRRSV samples were treated at 56–100°C for various times. Third, 50 μL distinct concentrations of bleach (300–500 mg/L), bromogeramine (2–4 g/L), and PCMX (2–10 g/L), were interacted with 200 μL PRRSV samples for 30 min at room temperature.

### 2.4. Immunofluorescence Assay (IFA)

IFA was executed in PAMs as we described previously [25]. The infected PAMs were collected at 72 hpi for IFA detection. Briefly, the infected PAMs were washed with PBS and then fixed with 4% paraformaldehyde. Cells were permeabilized with 0.5% TritonX-100 for 10 min and then blocked with 1% BSA for 2 h. PRRSV-specific murine mAb 15A1 (1:500) and Dylight 594 goat anti-mouse IgG (1:1000, Invitrogen, USA) were used as the primary and secondary antibodies, respectively [22]. The cells were observed with the IX53 inverted fluorescence microscope (Olympus, Japan).

### 2.5. Optimization of PMA Pretreatment

The PMA pretreatment parameters for discriminating live and inactivated viruses were also optimized [21–23]. In detail, inactivated PRRSV samples were pretreated with different concentrations (1–50 μM) of PMA (Biotium, United States) for various times (5–20 min) at different temperatures (0–37°C). Photolysis was executed directly under blue light (BL-12, Beijing Labgic Technology Co. Ltd.) for 5–20 min. And then, viral RNA was extracted with TRIpure Reagent (Aidlab, Beijing, China) and the cDNA was synthesized with PrimeScript 1st Strand cDNA Synthesis Kit (TaKaRa, Japan) as previously described [22].

### 2.6. PMA-qPCR Amplification

Three pairs of probes and primers (Table 1) used for the triplex PMA-qPCR were adapted and modified from our previous study [13, 25]. The concentrations of primers and probes were reoptimized to generate the best amplification for live virus and the lowest amplification for inactivated virus in the triplex PMA-qPCR detection system.

### 2.7. Statistical Analysis

Three replicates were used for each experiment carried out in this study. The viral RNA levels were presented by means±standard deviations (SD). The statistical analysis was executed using the Mann–Whitney *U* test [25].

## 3. Results

### 3.1. Inactivation of Prevalent PRRSV Isolates by Distinct Methods

**Figure 1:**
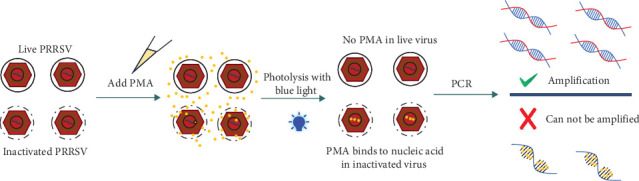
The procedures of PRRSV PMA-qPCR assay for differential detection. PMA cannot enter live PRRSV particle and do not affect PCR amplification. However, PMA can enter inactivated virus and bind to nucleic acid after photolysis with blue light, which will inhibit PCR amplification.

**Figure 2:**
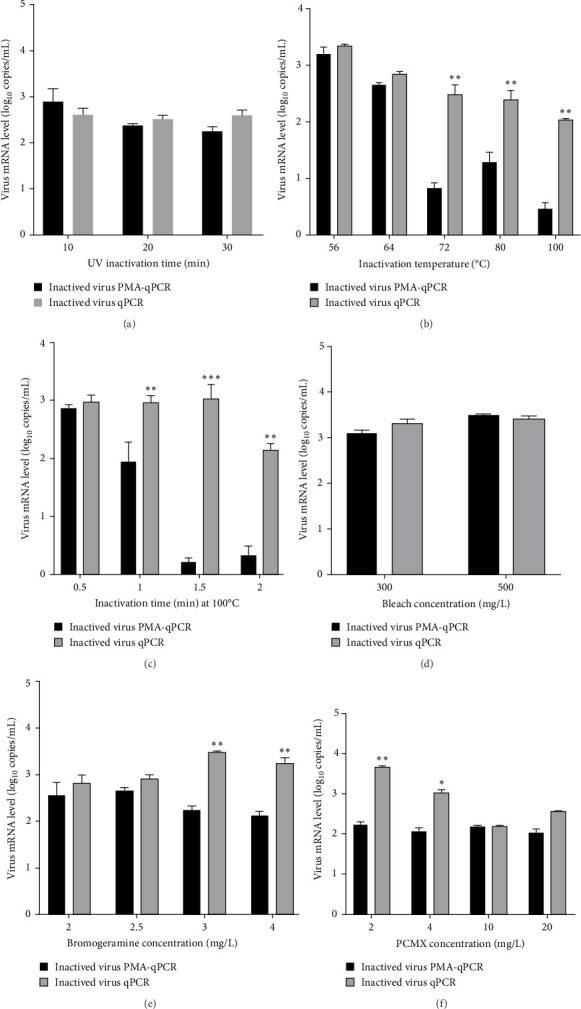
PRRSV inactivation efficacies by UV light, heat, and disinfectants. Same amount NADC34-like PRRSV-2 BJ1805-2 isolate (0.1 MOI) was used for all inactivation evaluations. The viral amplification efficacies were determined to assess inactivation efficacies. (A) PRRSV inactivation effects by exposure to UV light for 10–30 min. (B) PRRSV inactivation effects by heat from 56 to 100°C. (C) PRRSV inactivation effects by heat at 100°C for 0.5–2 min. (D) PRRSV inactivation effects by bleach. (E) PRRSV inactivation effects by bromogeramine. (F) PRRSV inactivation effects by PCMX. The statistical significance was set at *⁣*^*∗*^*p* < 0.05, *⁣*^*∗∗*^*p* < 0.01, and *⁣*^*∗∗∗*^*p* < 0.001.

**Figure 3:**
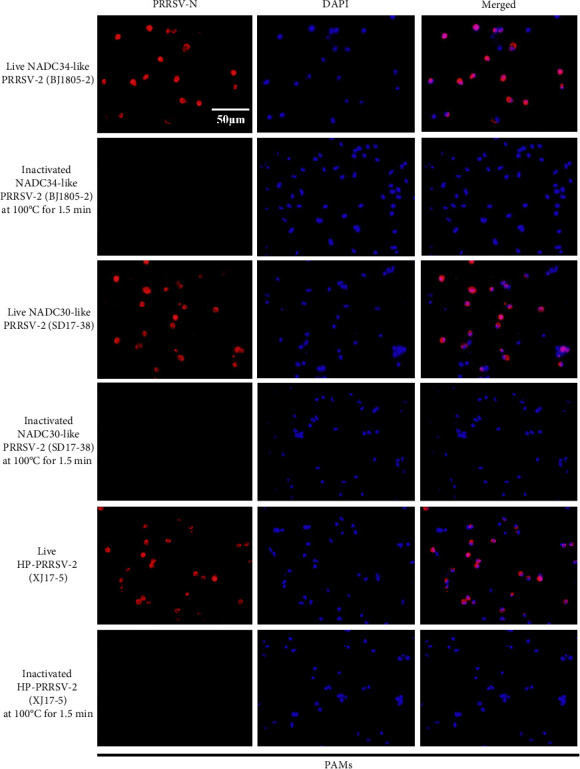
The inactivation efficacy was confirmed by virus inoculation and IFA detection. Live or inactivated (100°C 1.5 min) BJ1805-2, SD17-38, and XJ17-5 isolates (0.1 MOI) were used to infect PAMs and then examined by IFA at 72hpi.

**Figure 4:**
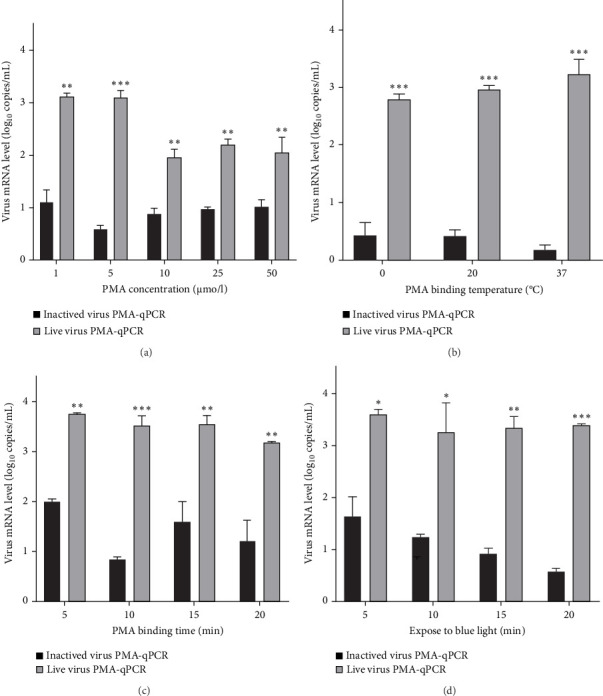
The optimization of PMA pretreatment parameters. (A) The influence of 1–50 μM PMA pretreatment on triplex PMA-qPCR amplification was determined. (B) The influence of PMA binding temperature (0–37°C). (C) The influence of PMA binding time (5–20 min). (D) The influence of photolysis time (exposure to blue light for 5–20 min). The statistical significance was set at *⁣*^*∗*^*p* < 0.05, *⁣*^*∗∗*^*p* < 0.01, and *⁣*^*∗∗∗*^*p* < 0.001.

To develop a PMA-qPCR assay for the discrimination of live and inactivated PRRSV (Figure 1), we first screened an effective PRRSV inactivation strategy by comparing the inactivation effects of UV light, heat and three disinfectants [22]. When treated with UV light (10–30 min), there was no significant difference between PMA-qPCR and qPCR amplifications (Figure 2A). When inactivated by heat (56–100°C), the biggest amplification difference was observed at 100°C for 1.5 min (*p* < 0.001) (Figure 2B,C). For the disinfectants, similar amplifications were noticed between PMA-qPCR and qPCR when NADC34-like PRRSV-2 (BJ1805-2) was inactivated with 300–500 mg/L bleach (Figure 2D). Noticeably, PMA-qPCR presented significantly lower amplification (*p* < 0.05) than qPCR when the virus was treated with 3–4 g/L bromogeramine (Figure 2E) and 2–4 g/L PCMX (Figure 2F). However, the highest difference in live and inactivated virus amplification was still at 100°C for 1.5 min. In addition, IFA detection in PAMs further confirmed that all representative PRRSV strains (NADC34-like BJ1805-2, NADC30-like SD17-38, and HP-PRRSV-2 XJ17-5) could be completely inactivated by heating at 100°C for 1.5 min (Figure 3). Therefore, 100°C 1.5 min was utilized for PRRSV inactivation in the optimization of the triplex PMA-qPCR assay.

### 3.2. PRRSV Triplex PMA-qPCR Optimization

NADC34-like PRRSV-2 BJ1805-2 live virus (0.1 MOI) or BJ1805-2 inactivated virus (100°C for 1.5 min) was utilized for the triplex PMA-qPCR optimization. When pretreated by 5 μM PMA, live BJ1805-2 virus had the highest viral RNA level, but the inactivated BJ1805-2 virus had the lowest viral RNA level (Figure 4A). Meanwhile, 37°C was determined as the best PMA binding temperature (Figure 4B). The most appropriate PMA binding time and photolysis time were 10 and 20 min, respectively (Figure 4C,D). The final PMA pretreatment was determined as: incubation of the viral sample with 5 μM PMA at 37°C for 10 min followed by photolysis with blue light for 20 min.

**Figure 5:**
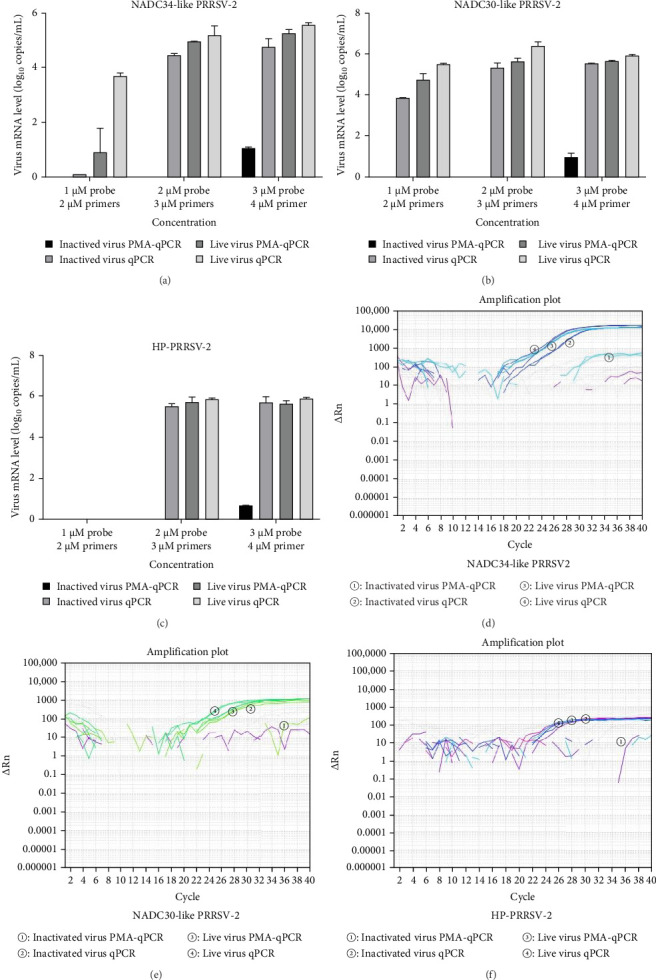
The influences of primer and probe concentrations on triplex PMA-qPCR amplification. (A–C) The influences of primer and probe concentrations on live and inactivated NADC34-like PRRSV-2, NADC30-like PRRSV-2, and HP-PRRSV-2 amplifications. (D–F) The amplifications of live and inactivated NADC34-like PRRSV-2, NADC30-like PRRSV-2, and HP-PRRSV-2 when using 2 μM probes and 3 μM primers.

To estimate the fine concentrations of primers and probes, 2–4 μM primers and 1–3 μM probes were utilized for triplex PMA-qPCR evaluation. Noticeably, viral RNAs could not be detected for all inactivated viruses (NADC34-like BJ1805-2, NADC30-like SD17-38, and HP-PRRSV-2 XJ17-5) when 3 μM primers and 2 μM probes were applied (Figure 5A–C). Meanwhile, the other groups (live virus without PMA pretreatment, live virus with PMA pretreatment, and inactivated virus without PMA pretreatment) have same levels of amplifications (Figure 5D–F). Therefore, 3 μM primers and 2 μM probes were utilized in the PRRSV triplex PMA-qPCR method, which was carried out in a 20 μL reaction system containing 2 μL cDNA, 0.3 μL primers (10 μM), 0.2 μL probes (10 μM), and 10 μL Premix Taq (TaKaRa, Japan). The amplification was performed at 95°C for 30 s, followed by 40 cycles of 95°C for 5 s and 60°C for 1 min. The fluorescence signal was collected at the end of the 60°C extension step in each cycle. When the Ct value was <35, it's considered positive. When the Ct value is ≥35 but <40, a retest is required. When the Ct value is ≥40, it's considered negative.

### 3.3. Estimation of the Triplex PMA-qPCR Method

**Figure 6:**
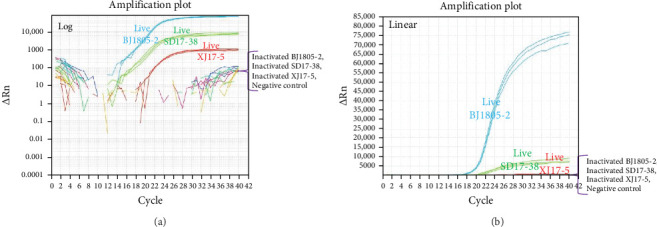
Discrimination of live and inactivated PRRSV isolates by this triplex PMA-qPCR assay. The live PRRSV samples including HP-PRRSV-2 (XJ17-5 isolate), NADC30-like PRRSV-2 (SD17-38 isolate), and NADC34-like PRRSV-2 (BJ1805-2 isolate) could be simultaneously detected while the corresponding inactivated viruses were undetectable. (A) Log image. (B) Linear image.

**Figure 7:**
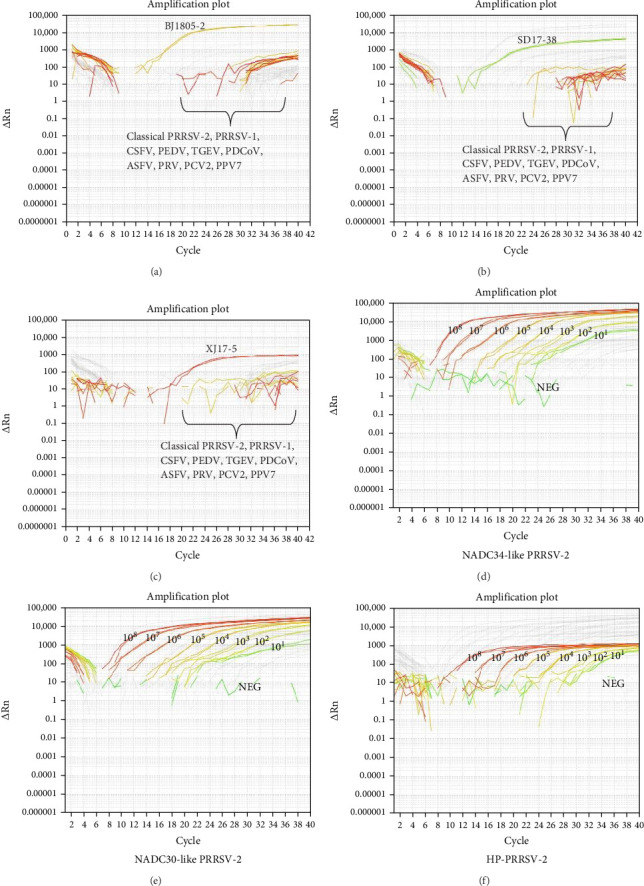
Specificity and sensitivity evaluations of the triplex PMA-qPCR assay. (A–C) FAM fluorescent signals were observed only when live NADC34-like BJ1805-2, NADC30-like SD17-38 and HP-PRRSV-2 XJ17-5 isolates were detected but not for other viruses. (D–F) The detection limits of this triplex PMA-qPCR assay were 6 × 10^1.0^ (60) copies/reaction for each virus, respectively.

Representative live and inactivated PRRSV isolates were used to assess the detection capacity of this new triplex PMA-qPCR assay. NADC34-like BJ1805-2, NADC30-like SD17-38, and HP-PRRSV-2 XJ17-5 live viruses could be simultaneously detected, while the corresponding inactivated viruses could not be determined by this triplex PMA-qPCR assay (Figure 6). Meanwhile, specificity evaluation showed that live PRRSV-1 SD1291 isolate, classical PRRSV-2 VR-2332-like JSYC20-05-1, and CH-1a-like SD1612-1 isolates, and other common porcine viruses (CSFV, PEDV, TGEV, PDCoV, ASFV, PRV, PCV2, and PPV7) could not be detected (Figure 7A–C). Furthermore, this triplex PMA-qPCR exhibited satisfied sensitivity with detection limits of 60 copies for each virus (Figure 7D–F). Moreover, the coefficients of variations (CVs) for intra-assay ranged from 0.25% to 3.36% and CVs for interassay varied from 0.52% to 4.49% (Table 2), indicating that this triplex PMA-qPCR assay also has tolerable reproducibility.

To evaluate the discrimination capacity of live and inactivated PRRSV by this triplex PMA-qPCR assay, the triplex PMA-qPCR was used to detect serial mixtures of distinct ratios of live and inactivated NADC34-like PRRSV-2 BJ1805-2 strain. The Ct values were produced by the triplex qPCR detection on four samples (100% Live BJ1805-2 virus, 10% live BJ1805-2 virus + 90% inactivated BJ1805-2 virus, 1% live BJ1805-2 virus + 99% inactivated BJ1805-2 virus, 100% inactivated BJ1805-2 virus) were quit similar (Table 3). Meanwhile, the between triplex qPCR and triplex PMA-qPCR on 100% live BJ1805-2 virus sample, 10% live BJ1805-2 virus + 90% inactivated BJ1805-2 virus, and 1% live BJ1805-2 virus + 99% inactivated BJ1805-2 virus were 0.42, 3.53, and 6.91 when detected by the triplex PMA-qPCR, respectively. The ΔCt values in the triplex PMA-qPCR were close to the ideal conditions (shown in brackets). More importantly, the 100% inactivated BJ1805-2 virus was undetectable in the triplex PMA-qPCR assay. The above results verified that this triplex PMA-qPCR assay can be used for the discrimination of live and inactivated PRRSV isolates prevalent in Chinese swine herds.

### 3.4. Clinical Evaluation of the Triplex PMA-qPCR and qPCR Assays

To validate this triplex PMA-qPCR assay, 452 clinical samples were detected by both triplex PMA-qPCR and triplex qPCR assays. There were 30 environmental feces samples (30 out of 113, 26.55%) were PRRSV-positive detected by the triplex qPCR, while only 22 samples (22/113, 19.47%) were positive tested by the triplex PMA-qPCR. In addition, 40 (40/278, 14.39%) and 31 (31/278, 11.15%) lungs were detected positive in triplex qPCR and triplex PMA-qPCR, respectively. Moreover, 6 (6/24, 25.00%) and 5 (5/24, 20.83%) sera were detected positive in triplex qPCR and triplex PMA-qPCR, while seven LN samples (7/37, 18.92%) were detected positive in both the triplex qPCR and PMA-qPCR. Overall, 83 samples (83/452, 18.36%) were detected as qPCR positive while 65 samples (65/452, 14.38%) were detected as PMA-qPCR positive (Table 4). In details, the triplex qPCR identified 25 NADC34-like PRRSV-2, 48 NADC30-like PRRSV-2, and 15 HP-PRRSV-2 positive samples, including two samples were coinfected by NADC34-like and NADC30-like PRRSV-2 and three samples were coinfected by NADC30-like and HP-PRRSV-2. Meanwhile, the triplex PMA-qPCR detected 21 NADC34-like PRRSV-2, 36 NADC30-like PRRSV-2, and 9 HP-PRRSV-2 positive samples, including one sample was coinfected by NADC34-like and NADC30-like PRRSV-2 (Table S1). The 18 qPCR positive but PMA-qPCR-negative feces samples were submitted to PRRSV isolation in PAMs, but no virus was isolated. However, distinct PRRSV strains could be isolated in representative PMA-qPCR-positive clinical samples (Figure S1).

**Figure 8:**
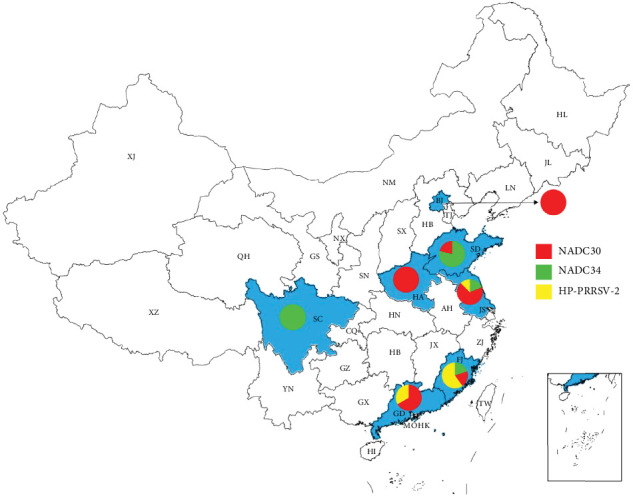
Geographical distribution of distinct PRRSV positive samples in China determined by triplex PMA-qPCR assay. There were 65 out of 452 clinical samples were detected positive by the triplex PMA-qPCR assay. The seven PRRSV-positive regions were shown in blue. The proportions of NADC34-like, NADC30-like, and HP-PRRSV-2 positive samples in each province were shown by a colored pie chart.

We further evaluated the geographical distribution of distinct PRRSV isolates in the 65 triplex PMA-qPCR-positive samples (Figure 8). In detail, the only one positive sample from Sichuan province was NADC34-like PRRSV-2. The four and 13 positive samples from Beijing City and Henan Province were all NADC30-like PRRSV-2. The three positive samples from Guangdong province contained 1 NADC34-like PRRSV-2 and 2 NADC30-like PRRSV-2 isolates. The 19 positive samples from Shandong provinces included 15 NADC34-like PRRSV-2 and 4 NADC30-like PRRSV-2 strains. The 10 positive samples from Fujian province contained 2 NADC34-like, 2 NADC30-like, and 6 HP-PRRSV-2 isolates. The 15 positive samples from Jiangsu province included 3 NADC34-like, 11 NADC30-like, and 2 HP-PRRSV-2 strains, along with 1 NADC34-like and NADC30-like PRRSV-2 coinfection. Overall, this newly developed triplex PMA-qPCR method could be used for differential detection of live PRRSV isolates in clinical samples.

## 4. Discussion

PRRSV has been prevalent in global swine herds for decades, which can be spread not only by direct contact but also by airborne transmission [27]. PRRSV detection is mainly relied on molecular biological methods such as RT-PCR [11], and serological method such as ELISA [28]. However, these commonly utilized methods cannot distinguish live and inactivated viruses. Virus isolation could be used to determine PRRSV infectivity, but it's time-consuming. In addition, PCR-positive clinical samples could not be used for PRRSV isolation when they only contained noninfectious viruses. More importantly, environmental samples containing PRRSV nucleic acid could be detected as positive by PCR even when the virus was inactivated, which will lead to a false positive diagnosis of an environmental contamination situation. Considering the prevalence of NADC34-like, NADC30-like, and HP-PRRSV-2 in Chinese swine herds [10, 29], a rapid differential detection method for these live viruses would be critical for PRRS control in China.

Previous studies have developed PMA-qPCR assays for several bacteria and viruses [30, 31]. For instance, the live and dead *Staphylococcus aureus*, *Salmonella app.*, and *Escherichia coli* could be discriminated by a multiplex PMA-qPCR assay [30]. SDS-PMA-assisted RT-qPCR might be used for specific determination of live SARS-CoV-2 in the PCR-positive samples [32]. Noticeably, their method did not really discriminate infectious and noninfectious SARS-CoV-2 but predicted the infectivity according to a cutoff ΔCt value (ΔCt ≥ 8.6). ASFV infectivity was also evaluated by PMA-based qPCR assays [23, 33]. Remarkably, these two ASFV PMA-qPCR assays utilized inconsistent disinfection and PMA pretreatment strategies. Accordingly, we proposed that the disinfection methods and PMA pretreatment conditions should be optimized for the development of distinct PMA-qPCR assays [22, 23, 32, 33]. Herein, we developed a PMA-based qPCR assay for differential detection of infectious PRRSV isolates prevalent in China.

At the beginning of this study, we selected an effective strategy for PRRSV inactivation. Although heat inactivation was determined to be not satisfied for ASFV inactivation [23, 33], heat at 100°C for 1.5 min is an effective inactivation strategy for distinct PRRSV isolates. The results were validated by virus isolation and IFA detection in PAMs. The distinct inactivation efficacies between ASFV and PRRSV are likely associated with the differences in architectures of viral particles [22, 34]. Our results also showed that UV light and bleach inactivated PRRSV may not be qualified for PMA-qPCR detection, while bromogeramine and PCMX inactivated PRRSV might be differentiated by our PMA-qPCR assay. These results would guide a more rational application of this new triplex PMA-qPCR assay in the field.

PMA concentration is an important factor affecting the establishment of PMA-qPCR assays. Previously, 30 μM PMA pretreatment was utilized for the discrimination of live and dead *Escherichia coli*, *Salmonella app.*, and *Staphylococcus aureus* [30]. In addition, 50 μM PMA pretreatment was used to determine SARS-CoV-2 infectivity [32]. Moreover, 100 μM PMA was applied to discriminate live and inactivated enteric viruses [35]. Overall, these previous studies suggested that 50–150 μM PMA is generally utilized for virus pretreatment and 4–25 μM PMA is commonly used for bacteria pretreatment [22, 31]. However, here we presented that 5 μM PMA pretreatment could effectively discriminate live and inactivated PRRSV isolates, which might be associated with the loose envelope structure of PRRSV. In addition, the other parameters, including PMA binding time temperature and exposure time to blue light, might also affect the PMA pretreatment effects. Therefore, all the above criteria needed to be re-optimized for each pathogen-specific PMA-qPCR assay.

This newly developed triplex PMA-qPCR assay presented several advantages when comparing with the gold standard assay (virus isolation). The application of this triplex PMA-qPCR assay on environmental feces not only could evaluate the spread of distinct PRRSV in pig herds but also could provide direct evidence on the inactivation efficacies of some disinfectants. In addition, rapid differential detection of infectious PRRSV isolates prevalent in China could be achieved within few hours. Therefore, it might facilitate the screening of qualified samples for virus isolation in the following studies. What's more, this triplex PMA-qPCR assay might also be used for quality control of PRRS vaccines. Whether commercial PRRS vaccines were contaminated by the prevalent PRRSV isolates could be alternatively determined using this triplex PMA-qPCR method. Remarkably, considering that feces collection is a non-invasive method reducing unnecessary human interference of pigs, the ability of using this triplex PMA-qPCR assay for fecal sample detection would largely facilitate large-scale surveillances of PRRSV infection and environmental contamination statuses in the pig farms.

However, this triplex PMA-qPCR assay also had some disadvantages. For instance, this assay focuses on three major PRRSV isolates prevalent in China. PRRSV-1 and classical PRRSV-2 isolates could not be detected by this assay. Therefore, the usage of this triplex PMA-qPCR assay should be combined with the universal PMA-qPCR assay for rapid detection and simultaneous differentiation of infectious PRRSV isolates [22]. In addition, when the virus was inactivated by UV light or bleach, it might not be eligible for discrimination by the PMA-qPCR assays. Another limitation of this study is about the representative of clinical samples. Even though 452 samples (including environmental feces, lungs, LNs, and sera) from seven regions of China (Jiangsu, Beijing, Fujian, Shandong, Guangdong, Henan, and Sichuan) collected from April 26th 2020 to February 21st 2025 were utilized, the detailed background, such as pig farm scale and infection status of the pigs was not provided by the farm owners. Therefore, more standard representative samples are still needed to comprehensively evaluate the efficiency of this new triplex PMA-qPCR assay in the following study.

In conclusion, this is the first report of a PRRSV triplex PMA-qPCR assay for the infectivity discrimination of three PRRSV isolates prevalent in China. This triplex PMA-qPCR can be utilized to monitor and differentiate PRRSV environmental contamination. In addition, it may be used in the laboratory for clinical sample screening. Moreover, it may also be applied to evaluate the qualities of commercial PRRS vaccines. Overall, this study provides an alternative and practical strategy for differential detection of infectious PRRSV isolates. A rational application of this triplex PMA-qPCR assay will facilitate PRRSV diagnosis, prevention, and control.

## Figures and Tables

**Table 1:** Primers and probes used in the triplex PMA-qPCR and triplex qPCR assays.

No.	Primer/probe	Sequence (5'-3')	Location
1	NADC34-F23	TGGTTGGCGTTCTTGTCCTT	ORF6
2	NADC34-P23	FAM-TGTGAGCACCGTTTAT-MGB	
3	NADC34-R23	CATCATGAACGGCACAAATGA	

4	NADC30-F23-2	TGGGGGGTGTATTCAGCCATG	ORF6
5	NADC30-P23-2	JOE-TTCATCCGATAACGGCAAG-MGB	
6	NADC30-R23-2	CGGACGACAAATGCGTGGTT	

7	HP-PRRSV2-F23	AGTGGGTCGGCACCAGTTC	NSP2
8	HP-PRRSV2-P23	TAMRA-CGTAGAACTGTGACAACAA -MGB	
9	HP-PRRSV2-R23	GCAGACAAATCCAGAGGCTCA	

*Note*: The primers and probes were modified from our previous study [13].

**Table 2:** Intrarepeatability and interreproducibility of this PRRSV PMA-qPCR assay.

Sample^a^	Intrarepeatability	Coefficient of variation (CV, %)	Interreproducibility	Coefficient of variation (CV, %)
10^6^ TCID^50^/ml	15.30 + 0.12^b^	0.25	15.45 ± 0.34	0.52
10^4^ TCID^50^/ml	21.78 ± 0.72	2.78	22.15 ± 0.85	3.03
10^2^ TCID^50^/ml	28.33 ± 0.95	3.36	28.88 ± 1.15	4.49

^a^Different amounts (10^2^~10^6^TCID^50^/ml) of NADC34-like PRRSV2 BJ1805-2 isolate were used.

^b^The mean of Ct ± standard deviation from three replicates.

**Table 3:** Triplex qPCRand triplex PMA-qPCR detections on serial mixtures of live and inactivated NADC34-likeBJ1805-2 virus.

Group	100% Live virus	10% live virus + 90% inactivated virus	1% live virus + 99% inactivated virus	100% inactivated virus
Triplex qPCR	17.56 ± 0.42	17.42 ± 0.71	17.72 ± 0.53	17.93 ± 0.65
Triplex PMA-qPCR	17.98 ± 0.33	20.95 ± 0.57	24.63 ± 0.69	—^b^
ΔCt	0.42 (0)^a^	3.53 (3.33)	6.91 (6.67)	—

^a^The ideal ΔCt values between triplex qPCR and triplex PMA-qPCR on Anheal-1 isolate were shown in the brackets.

^b^The slash indicates undetectable.

**Table 4:** Clinical sample information.

Sample type	Sample no.	Sample percentage (%)	qPCR positive no.^a^	qPCR positive percentage (%)	PMA-qPCR positive no.^b^ (%)	PMA-qPCR positive percentage (%)
Feces	113	25.00	30	26.55	22	19.47
Lung	278	61.50	40	14.39	31	11.15
LN	37	8.19	7	18.92	7	18.92
Serum	24	5.31	6	25.00	5	20.83
Total	452	100	83	18.36	65	14.38

^a^The results were determined by the PRRSV triplex qPCR assay.

^b^The results were determined by the PRRSV triplex PMA-qPCR assay.

## Data Availability

The data used to support the findings of this study are included within the article and supplemental files.

## Supporting Information

Supporting Information 1 Table S1. Comparison of triplex PMA-qPCR and triplex qPCR detection on PRRSV positive clinical samples.

Supporting Information 2 Figure S1. PRRSV isolations from representative clinical samples. Three qPCR and PMA-qPCR positive samples and three qPCR positive but PMA-qPCR negative samples (100 μL filtered supernatant from each sample homogenate) were selected and used for virus isolation in PAMs. IFA detection was performed at 72 hpi. PRRSV N protein specific signals could only be detected in three qPCR and PMA-qPCR positive samples. Anheal-1 infection (0.01 MOI) was set as positive control and RPMI-1640 was added to negative control.
