# Supplementary material for: A Triplex Propidium Monoazide (PMA) qPCR Assay Enables Rapid Discrimination of Live Porcine Reproductive and Respiratory Syndrome Viruses
Source: Transbound Emerg Dis. 2025 Nov 7;2025:7921675. doi: 10.1155/tbed/7921675 (PMC12618118; doi:10.1155/tbed/7921675)
Supplement: Supporting Information 1 — Table S1. Comparison of triplex PMA-qPCR and triplex qPCR detection on PRRSV positive clinical samples. [file 7921675.f1.doc]

Table S1. Comparison of triplex PMA-qPCR and triplex qPCR detection on PRRSV positive clinical samples.

| No. | Name | Sample | Virus | Ct | |
| --- | --- | --- | --- | --- | --- |
|  |  |  |  | PMA-qPCR | qPCR |
| 1 | JSNJ20-869 | Feces | NADC30 | ND* | 25.81 |
| 2 | JSNJ20-873 | Feces | NADC34 | 32.15 | 31.19 |
| 3 | FJFZ20-983 | Feces | HP | 30.25 | 23.17 |
| 4 | FJFZ20-984 | Feces | HP | ND | 25.62 |
| 5 | FJFZ20-985 | Feces | HP | 28.24 | 22.84 |
| 6 | FJFZ20-986 | Feces | HP | 29.29 | 23.18 |
| 7 | FJFZ20-987 | Feces | HP | 30.36 | 23.42 |
| 8 | FJFZ20-988 | Feces | HP | 26.73 | 23.31 |
| 9 | FJFZ2020-993 | Feces | HP | 31.07 | 26.6 |
| 10 | FJFZ2020-994 | Feces | HP | ND | 24.3 |
| 11 | FJFZ2020-995 | Feces | NADC30 | 29.6 | 27.15 |
| 12 | FJFZ2020-997 | Feces | NADC30 | 30.15 | 27.86 |
| 13 | FJFZ2020-1000 | Feces | NADC34 | 30.05 | 29.75 |
| 14 | FJFZ2020-1026 | Feces | HP | ND | 25.51 |
| 15 | FJFZ2020-1027 | Feces | NADC34 | 29.5 | 26.78 |
| 16 | SDZB20-1047 | Feces | NADC34 | 27.87 | 26.48 |
| 17 | SDZB20-1048 | Feces | NADC34 | ND | 27.63 |
| 18 | SDZB20-1049 | Feces | NADC30 | 25.13 | 23.86 |
| 19 | SDZB20-1050 | Feces | NADC34 | ND | 26.84 |
| 20 | SDZB20-1051 | Feces | NADC34 | 28.96 | 28.21 |
| 21 | SDZB20-1052 | Feces | NADC30/NADC34 | ND/ND | 27.04/28.02 |
| 22 | SDZB20-1053 | Feces | NADC34 | 27.83 | 27.07 |
| 23 | SDZB20-1054 | Feces | NADC34 | 31.03 | 28.15 |
| 24 | SDZB20-1055 | Feces | NADC30 | 27.59 | 25.61 |
| 25 | SDZB20-1057 | Feces | NADC30 | 26.83 | 24.18 |
| 26 | SDZB20-1058 | Feces | NADC30 | 27.75 | 20.8 |
| 27 | SDZB20-1060 | Feces | NADC34 | ND | 28.47 |
| 28 | SDZB20-1061 | Feces | NADC34 | 27.85 | 26.83 |
| 29 | SDZB20-1062 | Feces | NADC34 | 28.93 | 28.53 |
| 30 | SDZB20-1064 | Feces | NADC34 | 29.48 | 26.45 |
| 31 | SDZB20-1065 | LN | NADC34 | 29.58 | 27.75 |
| 32 | JSYC21-1245 | LN | NADC34 | 29.80 | 27.54 |
| 33 | SDLY23-1734 | Lung | NADC34 | 27.52 | 21.75 |
| 34 | SDLY23-1735 | Lung | NADC34 | 28.73 | 23.17 |
| 35 | SDLY23-1738 | Lung | NADC34 | 28.25 | 23.31 |
| 36 | SDLY23-1739 | Lung | NADC34 | 31.55 | 25.17 |
| 37 | SDLY23-1742 | Lung | NADC34 | 21.42 | 17.36 |
| 38 | SDLY23-1743 | Lung | NADC34 | 28.45 | 25.58 |
| 39 | SDLY23-1744 | Lung | NADC34 | 27.55 | 23.02 |
| 40 | SCNJ23-1875 | Lung | NADC34 | 27.58 | 25.82 |
| 41 | GDCZ23-2122 | Serum | NADC30 | 31.22 | 29.43 |
| 42 | GDCZ23-2637 | LN | NADC30 | 27.57 | 20.92 |
| 43 | BJ23-2663 | Lung | NADC30 | 25.53 | 23.38 |
| 44 | JSYZ23-2667 | Lung | NADC30/NADC34 | 27.45/26.72 | 24.02/24.55 |
| 45 | JSYZ23-2668 | Lung | HP | 30.99 | 27.47 |
| 46 | JSYZ23-2670 | Lung | HP | 30.04 | 26.55 |
| 47 | JSYZ24-2706 | Serum | NADC30 | 30.23 | 29.96 |
| 48 | JSYZ24-2707 | Serum | NADC30 | 31.68 | 30.54 |
| 49 | HNZK24-2708 | Lung | NADC30 | 29.35 | 21.31 |
| 50 | HNZK24-2709 | LN | NADC30 | 29.07 | 26.58 |
| 51 | HNZK24-2710 | LN | NADC30 | 23.89 | 22.15 |
| 52 | HNZK24-2711 | LN | NADC30 | 22.15 | 17.65 |
| 53 | JSYZ24-2750 | Serum | NADC30 | 34.06 | 30.13 |
| 54 | JSYZ24-2751 | Serum | NADC30 | 30.90 | 30.72 |
| 55 | JSYZ24-2752 | Serum | NADC30 | ND | 31.91 |
| 56 | JSYZ24-2760 | LN | NADC30 | 24.52 | 21.23 |
| 57 | GDST24-2798 | Lung | NADC30/HP | ND/ND | 27.21/25.19 |
| 58 | GDST24-2799 | Lung | NADC30/HP | ND/ND | 32.85/31.24 |
| 59 | GDST24-2800 | Lung | HP | 16.83 | 18.69 |
| 60 | GDST24-2801 | Lung | NADC30 | ND | 30.73 |
| 61 | GDST24-2802 | Lung | NADC30 | ND | 30.75 |
| 62 | JSYZ24-2813 | Lung | NADC30 | ND | 29.43 |
| 63 | JSYZ24-2815 | Lung | NADC30 | 18.94 | 19.92 |
| 64 | JSYZ24-2823 | Lung | NADC30 | 26.53 | 24.94 |
| 65 | JSYZ24-2824 | Lung | NADC30 | 18.25 | 16.17 |
| 66 | JSYZ24-2825 | Lung | NADC30 | 26.11 | 21.76 |
| 67 | BJ24-2838 | Lung | NADC30 | 26.77 | 26.55 |
| 68 | BJ24-2842 | Lung | NADC30 | 29.79 | 24.97 |
| 69 | BJ24-2843 | Lung | NADC30 | 31.26 | 16.97 |
| 70 | JSYZ24-2845 | Lung | NADC30 | 30.03 | 27.44 |
| 71 | JSYZ25-2847 | Lung | NADC30 | ND | 30.24 |
| 72 | JSYZ25-2848 | Lung | NADC30 | ND | 29.94 |
| 73 | HNZMD25-2852 | Lung | NADC30 | 31.16 | 27.32 |
| 74 | HNZMD25-2856 | Lung | NADC30 | 32.78 | 28.59 |
| 75 | HNZMD25-2859 | Lung | NADC30 | 31.89 | 29.33 |
| 76 | HNZMD25-2860 | Lung | NADC30 | 28.90 | 25.48 |
| 77 | HNZMD25-2861 | Lung | NADC30 | 30.18 | 25.33 |
| 78 | HNZMD25-2862 | Lung | NADC30 | 32.75 | 29.09 |
| 79 | HNZMD25-2865 | Lung | NADC30 | 28.75 | 26.81 |
| 80 | HNZMD25-2867 | Lung | NADC30 | 31.07 | 26.12 |
| 81 | HNZMD25-2868 | Lung | NADC30 | 29.75 | 27.17 |
| 82 | HNZMD25-2869 | Lung | NADC30 | ND | 31.13 |
| 83 | GDST25-2884 | Lung | NADC30/HP | ND/ND | 31.70/29.17 |

* ND indicates not detectable.
