# Supplementary material for: A Triplex Propidium Monoazide (PMA) qPCR Assay Enables Rapid Discrimination of Live Porcine Reproductive and Respiratory Syndrome Viruses
Source: Transbound Emerg Dis. 2025 Nov 7;2025:7921675. doi: 10.1155/tbed/7921675 (PMC12618118; doi:10.1155/tbed/7921675)
Supplement: Supporting Information 2 — Figure S1. PRRSV isolations from representative clinical samples. Three qPCR and PMA-qPCR positive samples and three qPCR positive but PMA-qPCR negative samples (100 μL filtered supernatant from each sample homogenate) were selected and used for virus isolation in PAMs. IFA detection was performed at 72 hpi. PRRSV N protein specific signals could only be detected in three qPCR and PMA-qPCR positive samples. Anheal-1 infection (0.01 MOI) was set as positive control and RPMI-1640 was added to negative control. [file 7921675.f2.pptx]

## Slide 1
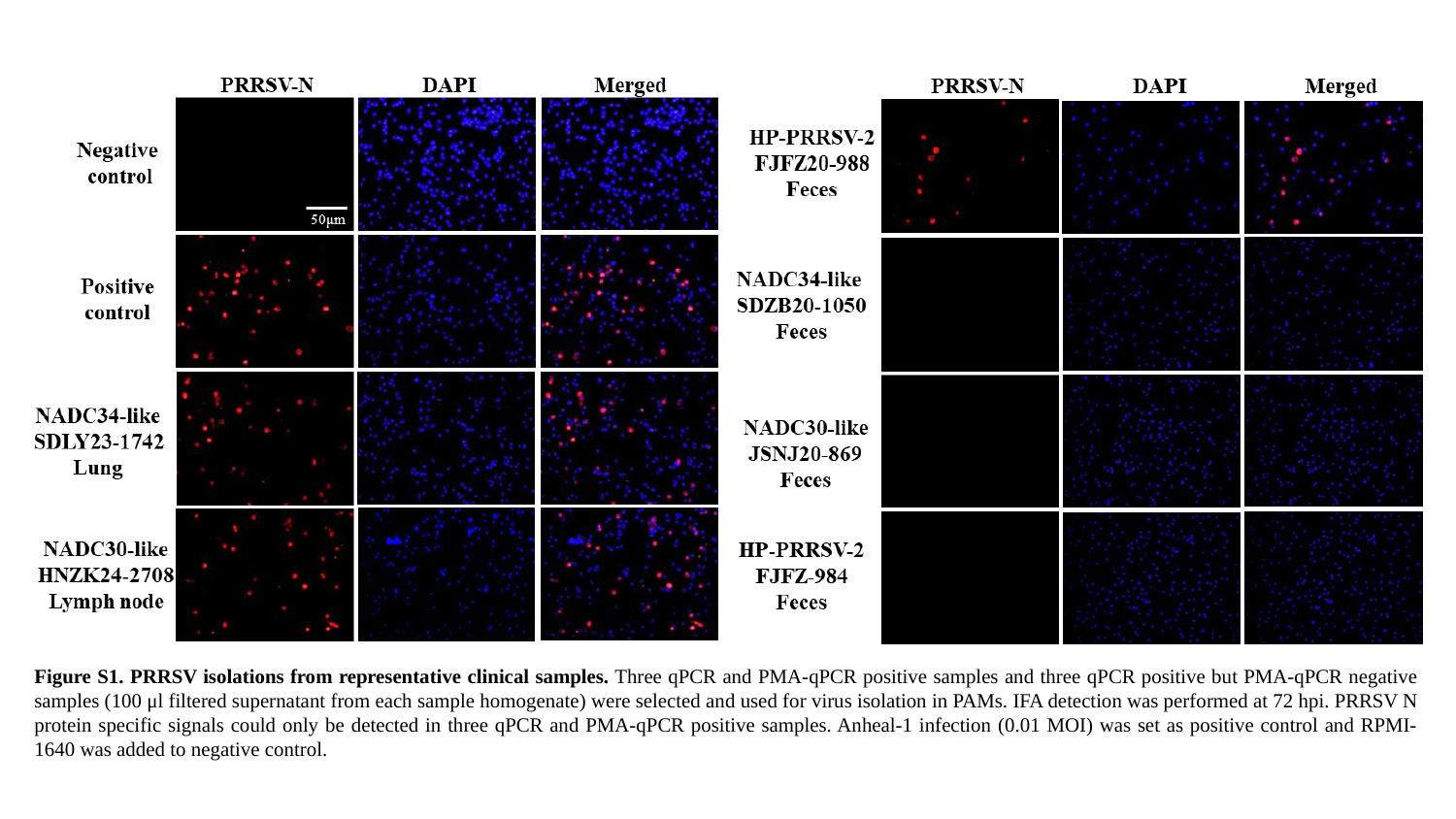

Figure S1. PRRSV isolations from representative clinical samples. Three qPCR and PMA-qPCR positive samples and three qPCR positive but PMA-qPCR negative samples (100 μl filtered supernatant from each sample homogenate) were selected and used for virus isolation in PAMs. IFA detection was performed at 72 hpi. PRRSV N protein specific signals could only be detected in three qPCR and PMA-qPCR positive samples. Anheal-1 infection (0.01 MOI) was set as positive control and RPMI-1640 was added to negative control.
